# Areca nut is associated with younger age of diagnosis, poor chemoradiotherapy response, and shorter overall survival in esophageal squamous cell carcinoma

**DOI:** 10.1371/journal.pone.0172752

**Published:** 2017-02-28

**Authors:** Chang-Han Chen, Hung-I Lu, Yu-Ming Wang, Yen-Hao Chen, Chien-Ming Lo, Wan-Ting Huang, Shau-Hsuan Li

**Affiliations:** 1 Institute for Translational Research in Biomedicine, Kaohsiung Chang Gung Memorial Hospital and Department of Applied Chemistry, and Graduate Institute of Biomedicine and Biomedical Technology, National Chi-Nan University, Taiwan; 2 Department of Thoracic & Cardiovascular Surgery, Kaohsiung Chang Gung Memorial Hospital and Chang Gung University College of Medicine, Kaohsiung, Taiwan; 3 Department of Radiation Oncology, Kaohsiung Chang Gung Memorial Hospital and Chang Gung University College of Medicine, Kaohsiung, Taiwan; 4 Department of Hematology-Oncology, Kaohsiung Chang Gung Memorial Hospital and Chang Gung University College of Medicine, Kaohsiung, Taiwan; 5 Department of Pathology, Kaohsiung Chang Gung Memorial Hospital and Chang Gung University College of Medicine, Kaohsiung, Taiwan; Emory University Winship Cancer Institute, UNITED STATES

## Abstract

**Objective:**

Areca nut chewing is carcinogenic to humans. However, little is known about the impact of areca nut chewing on esophageal squamous cell carcinoma (ESCC).

**Methods:**

We retrospectively reviewed 286 ESCC patients who received surgery or preoperative chemoradiotherapy followed by surgery at our institution. Background characteristics including areca nut chewing history were analyzed. The 4-nitroquinoline 1-oxide (4-NQO)-induced murine ESCC model was used to test the impact of arecoline, a main constituent of areca nut, on ESCC.

**Results:**

Compared to patients without areca nut chewing history, patients with areca nut chewing history had overall a younger age of onset (Mean age: 56.75 versus 52.68 yrs, P<0.001) and significantly worse overall survival than those without areca nut chewing history (P = 0.026). Among patients who received surgery, the overall survival rates were not significantly different between those with or without areca nut chewing history. Among patients who received preoperative chemoradiotherapy followed by surgery, those with areca nut chewing history had a significantly lower pathologic complete response rate (P = 0.002) and lower overall survival rate (P = 0.002) than those without. In the murine ESCC model, the incidence of esophageal invasive squamous cell carcinoma was 40% in mice exposed to concomitant 4-NQO and arecoline treatment for 8 weeks and 6% in mice exposed to 4-NQO only for 8 weeks (P = 0.037).

**Conclusions:**

Our results indicate that areca nut chewing history is significantly associated with younger age of onset, poor response to chemoradiotherapy, and shorter overall survival in ESCC patients. Arecoline, a main constituent of areca nut, accelerates esophageal tumorigenesis in the 4-NQO-induced murine ESCC model.

## Introduction

Esophageal cancer is one of the most prevalent cancers worldwide.[1] The most common histological types of esophageal cancer are squamous cell carcinoma and adenocarcinoma, and they constitute more than 90% of esophageal malignancies. These two major histological types of esophageal cancer have different pathogenesis, epidemiology, tumor biology and prognosis,[2] and thus should be analyzed separately. In Asia, the majority of esophageal cancers are esophageal squamous cell carcinoma (ESCC).[3] Despite significant improvements that have been made in surgical technique, chemotherapy, and radiotherapy, the prognosis of the patients with ESCC remains poor. The 5-year survival rate of patients with ESCC is around 20~30%.[4]

Tobacco smoking and high-alcohol intake are well-known risk factors for ESCC.[3] The habit of chewing areca nut is widespread in southern Asia and the South Pacific Islands, and it is estimated that there are approximately 600 million chewers around the world.[5] In Taiwan, more than 2 million people use areca nut, with the life-time prevalence being as high as 15%, and with more users among men than women (9.8% vs. 1.6%).[6] From epidemiological and animal studies, it is well-recognized that areca nut is an independent factor in the development of oral cancer.[7] Areca nut has been classified as a group 1 carcinogen by the International Agency for Research on Cancer.[8] Recently, several epidemiological studies and meta-analyses have shown that areca nut chewing is also a risk factor for ESCC.[9] However, little is known about the impact of areca nut chewing on ESCC. The aim of the present study was to investigate the relationship between areca nut chewing and the age of onset and treatment outcomes of major treatment modalities for ESCC.

## Methods

### Patient population

We retrospectively reviewed 286 patients with ESCC who received an esophagectomy at Kaohsiung Chang Gung Memorial Hospital. This study was approved by the Institutional Review Board of Chang Gung Memorial Hospital. Patients undergoing surgery had a radical esophagectomy with cervical esophagogastric anastomosis (McKeown procedure) or an Ivor Lewis esophagectomy with intrathoracic anastomosis, reconstruction of the digestive tract with a gastric tube, and pylorus drainage procedures. Two-field lymph node dissection was performed in all patients. For patients receiving preoperative chemoradiotherapy followed by surgery, the treatment protocol of the chemoradiotherapy was performed as previously described.[10] Briefly, two cycles of chemotherapy were performed concurrently with radiotherapy, and the chemotherapy regimen consisted of cisplatin (75~100 mg/m^2^; 4-hour drip) on day 1 and 5-fluorouracil (800~1000mg/m^2^; continuous infusion) on days 1–4 every 4 weeks. Three-dimensional conformal radiotherapy via a four-field technique, two parallel opposed anterio-posterier fields, one right posterior oblique field, and one left posterior oblique field, was used for most of the patients. The gross tumor volume (GTV) was defined as the gross tumor and lymph nodes based on the CT scan or positron emission tomography (PET) scan, if available. The clinical target volume (CTV) covered the GTV with a 3-cm craniocaudal and 1-cm radial margin and also the entire mediastinal lymph nodes. For upper or lower third primary tumors, bilateral supraclavicular lymph nodes or celiac lymph nodes were also included for prophylactic irradiation. The planning target volume (PTV) was generated from the CTV with a 1-cm expansion in all directions. The radiotherapy was delivered by LINAC using 6- to 15-MV photons with 1.8–2 Gy per daily fraction, five fractions per week. Before 2009, the total dose prescribed to the PTV was 36Gy in 18–20 daily fractions; by 2009, we changed our protocol for preoperative chemoradiotherapy followed by surgery and the total dose to the PTV was increased to 50–50.4 Gy in 25–28 fractions. Pathologic complete response (pCR) was defined as the complete disappearance of all viable cancer cells in all surgical specimens including the primary esophageal tumor and lymph nodes. The TNM stage was determined according to the 7^th^ American Joint Committee on Cancer (AJCC) staging system.[11] Overall survival (OS) was calculated from the date of diagnosis until the date of death or last follow-up. The medical histories of the patients, including their smoking, alcohol, and areca nut chewing habits were obtained from their medical records. Subjects who had consumed an alcoholic beverage ≧1 times per week for at least six months were considered to have a habit of alcohol consumption. Subjects who had smoked ≧10 tobacco cigarettes per week for at least six months were regarded as smokers. Subjects who had chewed ≧1 quid of areca nut per day for at least six months were included in the group of areca nut chewers.[12, 13]

### 4-nitroquinoline 1-oxide (4-NQO)-induced murine ESCC model

Six-week-old male C57BL/6 mice purchased from BioLasco (Taipei, Taiwan) were used in the present study. Our research team monitored animals once daily. Health was monitored by food and water intake, lethargy and inactivity, shallow or labored breathing, hair coat condition, hunched posture, impaired ambulation and change in the body weight and no unexpected death was found. All experimental procedures were approved by Institutional Animal Care and Use Committee (IACUC) of Kaohsiung Chang Gung Memorial Hospital. All efforts were made to minimize suffering to animals. During the conduct of animal experiment, animals that lost greater than 20% body weight, and interfered with the general health of the animal were euthanized prior to the completion of the study. Euthanasia was used in the animal experiment to minimized animal suffering and distress. Animals were euthanized by CO_2_ inhalation. We used 70% CO_2_ for 40 sec to terminate life of animals that reached humane endpoints. The 4-NQO (Sigma–Aldrich, St. Louis, MO, USA) and arecoline hydrobromide (Sigma–Aldrich, St. Louis, MO, USA) were dissolved in the drinking water. The six-week-old mice were randomized into one of six groups: group 1 mice (n = 16) received 4-NQO (100μg /mL) for 16 weeks and then only drinking water for another 12 weeks (total 28 weeks); group 2 mice (n = 16) received both 4-NQO (100μg /mL) and arecoline (500μg/mL) for 16 weeks and then only drinking water for another 12 weeks (total 28 weeks); group 3 mice (n = 16) received 4-NQO (100μg /mL) for 8 weeks and then only drinking water for another 20 weeks (total 28 weeks); group 4 mice (n = 16) received both 4-NQO (100μg /mL) and arecoline (500μg/mL) for 8 weeks and then only drinking water for another 20 weeks (total 28 weeks); group 5 mice (n = 8) received arecoline (500μg/mL) for 16 weeks and then only drinking water for another 12 weeks (total 28 weeks); group 6 mice (n = 8) received only drinking water for 28 weeks. Mice dying prior to the end of the experiment were excluded from the analysis. At the end of the experiment, mice were subjected to autopsies; whole esophagus and stomach were opened longitudinally, and macroscopic lesions were observed and identified carefully. The histological determination were performed by two pathologists (S.L.W. and W.T.H) according to the criteria described previously.[14] Hyperplasia was defined as thickened epithelium with prominent surface keratinization and with or without elongated rete ridges. Dysplasia was defined as loss of polarity in the epithelial cells, nuclear pleomorphism and hyperchromasia, abnormal single cell keratinization (dyskeratosis), and increased or abnormal mitoses. Papilloma was defined as noninvasive exophytic growth of neoplastic cells, and invasive squamous cell carcinoma was defined as a lesion with invasion into the subepithelial tissues.

### Immunohistochemistry

Immunohistochemical staining in human ESCC samples was performed by using standard reagents and techniques on a Bond-Max Automated Staining System (Leica Biosystems Newcastle Ltd, Australia). The sections were incubated with primary antibodies followed by Bond Polymer Refine detection system (DS9800, Leica Biosystems, Newcastle upon Tyne, UK). The primary antibody used was p53 (clone DO7, 1:200; Leica Biosystems, Newcastle upon Tyne, UK). Positive and negative controls were performed according to manufacturer’s instruction.. The staining assessment was independently carried out by 2 pathologists (S.L.W. and W.T.H) without any information about clinicopathologic features or prognosis. The p53 expression in the tissue was scored in terms of the percentage of cells that exhibited positive nuclear staining.

### Statistical analysis

Statistical analysis was performed using the SPSS 17 software package. For patient data, the chi-square test, Fisher’s exact test, or t-test was used to compare data between the two groups. Multivariate analysis of the response of induction chemotherapy was performed by logistic regression, and all variables were entered into the model. For survival analysis, the Kaplan–Meier method was used for univariate analysis, and the difference between survival curves was tested by a log-rank test. All parameters were entered into Cox regression model to analyze their relative prognostic importance. For mice experiments, statistical analyses of the incidence of esophageal lesions were performed using Chi-square or Fisher's exact test. For all analyses, a P value < 0.05 was considered statistically significant.

## Results

### Patient characteristics

The median age for the 286 patients (279 men and 7 women) was 53 years (range, 29–80). The 7th AJCC stages of 286 patients with ESCC were stage I in 58 patients, stage II in 65, and stage III in 163. The tumor locations were upper esophagus in 57 patients, middle in 115, and lower in 115. The histological grading were grade 1 in 41 patients, grade 2 in 185, and grade 3 in 60. Of the 286 patients, 262 (92%) reported habitual alcohol drinking, 254 (89%) cigarette smoking, and 184 (64%) areca nut chewing. Among the 286 patients, 129 patients received surgery, while 157 patients received preoperative chemoradiotherapy followed by surgery. At the time of analysis, the median periods of follow-up were 63 months for the 109 survivors, and 25 months for all 286 patients. The 3-year OS rate of these 286 patients was 45%.

The relationships between specific patient characteristics and the history/non-history of areca nut chewing and the different treatment modalities are shown in Table 1. Patients with areca nut chewing history had a significantly younger age of onset than those without areca nut chewing history (52.68-year-old versus 56.75-year-old; P<0.001). In addition, a history of areca nut chewing was associated with being male, a history of smoking, and a history of alcohol drinking. No other significant differences were seen between the patients with or without a history of areca nut chewing.

**Table 1:** Associations between areca nut chewing and clinicopathologic parameters in 286 patients with esophageal squamous cell carcinoma receiving surgery or preoperative chemoradiotherapy followed by surgery.

Parameters		All patients			Surgery			Preoperative CRT followed by surgery		
		Areca nut chewing		P value	Areca nut chewing		P value	Areca nut chewing		P value
		Absence (n = 102)	Presence (n = 184)		Absence (n = 50)	Presence (n = 79)		Absence (n = 52)	Presence (n = 105)	
Age (years, mean ± SD)		56.75±10.22	52.68±7.88	**0.001**	58.55±10.86	53.92±7.54	**0.01**	55.02±9.34	51.75±8.03	**0.034**
Sex	Male	96	183	**0.009**	47	78	0.30	49	105	**0.035**
	Female	6	1		3	1		3	0	
7^th^ AJCC stage	I/II	47	76	0.44	35	57	0.79	12	19	0.46
	III	55	108		15	22		40	86	
T classification	T1/2	34	65	0.73	30	53	0.41	4	12	0.47
	T3/4	68	119		20	26		48	93	
N classification	N0	41	73	0.93	30	55	0.26	11	17	0.44
	N1/2/3	61	111		20	24		41	88	
Tumor grade	Grade 1/2	81	145	0.90	41	59	0.33	40	84	0.66
	Grade 3	21	39		9	20		12	21	
Location	Middle/Lower	84	145	0.47	42	62	0.44	42	83	0.80
	Upper	18	39		8	17		10	22	
Smoking	Absence	25	7	**<0.001**	16	3	**<0.001**	9	4	**0.01**
	Presence	77	177		34	76		43	101	
Alcohol	Absence	19	5	**<0.001**	14	3	**<0.001**	5	2	**0.04**
	Presence	83	179		36	76		47	103	

CRT, chemoradiotherapy;

### Survival and areca nut chewing

**Fig 1:**
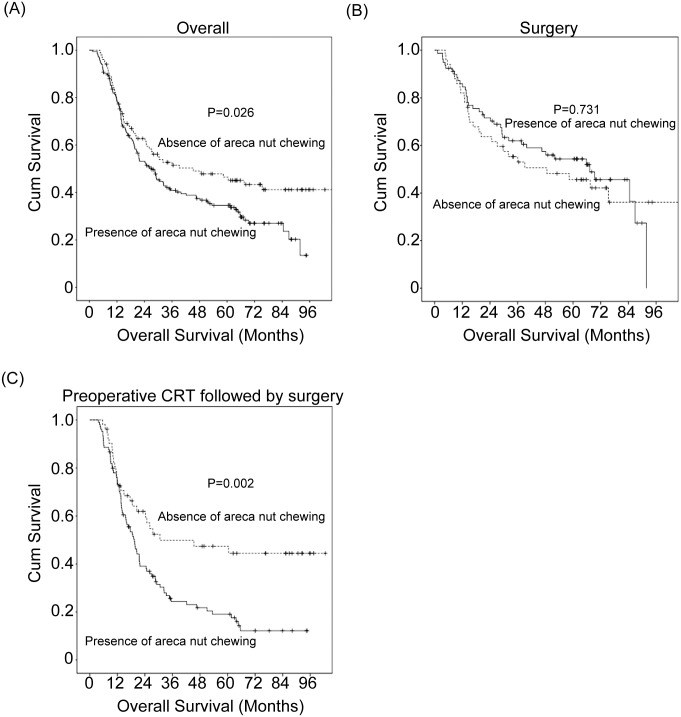
Kaplan–Meier survival curves according to areca nut chewing. (A) Overall patients. (B) Patients receiving surgery. (C) Patients receiving preoperative chemoradiotherapy followed by surgery.

Among all 286 patients, univariate analyses demonstrated that a history of areca nut chewing was significantly associated with the inferior OS (Table 2). The 3-year OS rate was 41% in patients with a history of areca nut chewing, compared with 53% in patients without a history of areca nut chewing (P = 0.026, Fig 1A). In multivariate comparison, 7^th^ AJCC stage III (P<0.001, odds ratio = 2.312, 95% confidence interval: 1.687–3.170) was independently associated with poorer OS. The areca nut chewing history showed a borderline significant trend (P = 0.055, odds ratio = 1.370, 95% confidence interval: 0.993–1.890).

**Table 2:** Results of univariate log-rank analysis of prognostic factors for overall survival in 286 patients with esophageal squamous cell carcinoma receiving surgery or preoperative chemoradiotherapy followed by surgery.

Parameters		All patients (n = 286)			Surgery (n = 129)			Preoperative CRT followed by surgery (n = 157)		
		No. of patients	3-year OS (%)	P value	No. of patients	3-year OS (%)	P value	No. of patients	3-year OS (%)	P value
Age	<53y/o	129	46%	0.97	52	69%	0.22	77	31%	0.59
	≧53y/o	157	44%		77	53%		80	34%	
7^th^ AJCC stage	I/II	123	65%	**<0.001**	92	73%	**<0.001**	31	42%	0.17
	III	163	29%		37	27%		126	30%	
T classification	T1/2	99	63%	**<0.001**	83	70%	**0.005**	16	34%	0.95
	T3/4	187	36%		46	41%		141	33%	
N classification	N0	114	67%	**<0.001**	85	75%	**<0.001**	29	41%	0.38
	N1/2/3	172	30%		44	29%		128	30%	
Tumor grade	Grade 1/2	226	47%	0.17	100	62%	0.20	126	33%	0.50
	Grade 3	60	39%		29	50%		31	29%	
Location	Middle/Lower	229	50%	**0.001**	104	65%	**<0.001**	125	36%	0.33
	Upper	57	27%		25	36%		32	21%	
Areca nut	Absence	102	53%	**0.026**	50	55%	0.73	52	50%	**0.002**
	Presence	184	41%		79	62%		105	24%	
Smoking	Absence	32	55%	0.095	19	47%	0.73	13	67%	**0.009**
	Presence	254	44%		110	62%		144	29%	
Alcohol	Absence	24	58%	0.12	17	53%	0.65	7	71%	0.087
	Presence	262	44%		112	60%		150	31%	

CRT, chemoradiotherapy;

### Survival and areca nut chewing according to the treatment method

For the 129 patients receiving surgery, we did not observe a significant association between a history of areca nut chewing with inferior OS (P = 0.73, Fig 1B).

For the 157 patients receiving preoperative chemoradiotherapy followed by surgery, univariate analyses found that areca nut chewing history and smoking history were significantly associated with inferior OS (Table 2). The 3-year OS rate was 24% in patients with areca nut chewing history, compared with 50% in patients without such history (P = 0.002, Fig 1C). In multivariate comparison, areca nut chewing history (P = 0.013, odds ratio = 1.770, 95% confidence interval: 1.128–2.776) and smoking history (P = 0.046, odds ratio = 2.533, 95% confidence interval: 1.016–6.318) remained independently associated with worse OS.

### The response to preoperative chemoradiotherapy and areca nut chewing

Because previous studies[15] showed that pCR to preoperative chemoradiotherapy predicts significantly improved survival, we tested whether substance use history is associated with the response to preoperative chemoradiotherapy. The relationships between the clinicopathological parameters and the response to preoperative chemoradiotherapy are summarized in Table 3. Absence of areca nut chewing history (P = 0.002) and absence of smoking history (P = 0.002) were significantly associated with pCR after preoperative chemoradiotherapy. The pCR rate was 44% in patients without areca nut chewing history compared with 21% in patients with such history. The logistic model showed that absence of areca nut chewing history (P = 0.018, odds ratio = 2.483, 95% confidence interval: 1.171–5.266) and absence of smoking history (P = 0.013, odds ratio = 5.073, 95% confidence interval: 1.417–18.158) were independently correlated with pCR after preoperative chemoradiotherapy.

**Table 3:** Associations between pathologic complete response and clinicopathological parameters in 157 patients with esophageal squamous cell carcinoma receiving preoperative chemoradiotherapy followed by surgery.

Parameters		Pathologic complete response		
		Presence	Absence	P value
Age	<53y/o	17	60	0.073
	≧53y/o	28	52	
7^th^ AJCC stage	I+II	10	21	0.62
	III	35	91	
T classification	T1+2	4	12	1.00
	T3+4	41	100	
N classification	N0	9	19	0.65
	N1+2+3	36	93	
Tumor grade	1+2	38	86	0.29
	3	7	26	
Tumor location	Middle/Lower	37	88	0.61
	Upper	8	24	
Areca nut chewing	Absence	23	29	**0.002**
	Presence	22	83	
Smoking	Absence	9	4	**0.002**
	Presence	36	108	

Chi-square test, or Fisher’s exact test was used for statistically analysis.

### The impact of arecoline on 4-NQO-induced murine ESCC model

**Fig 2:**
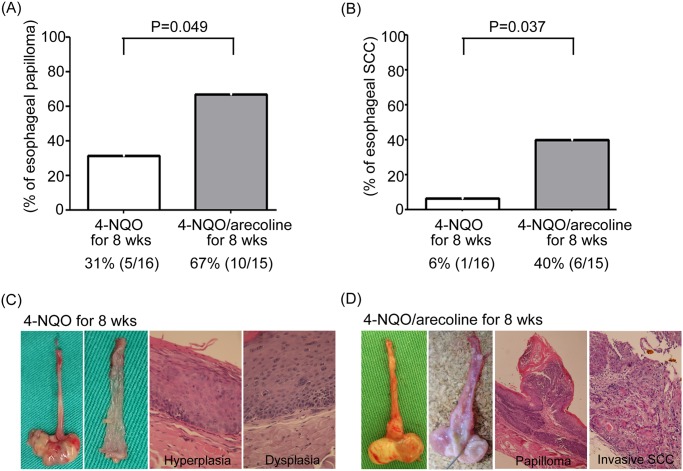
(A) The incidence of esophageal papilloma in mice exposed to concomitant 4-NQO and arecoline treatment for 8 weeks was significantly higher than that in mice exposed to 4-NQO only for 8 weeks (67% versus 31%; P = 0.049). (B) The incidence of esophageal invasive squamous cell carcinoma in mice exposed to concomitant 4-NQO and arecoline treatment for 8 weeks was also significantly higher than that in mice exposed to 4-NQO only for 8 weeks (40% versus 6%; P = 0.037). (C) Gross appearance of esophagus from representative mice exposed to 4-NQO only for 8 weeks. Hematoxylin and eosin stained (H&E) sections showed only esophageal hyperplasia and dysplasia. (D) Gross appearance of esophagus from representative mice exposed to concomitant 4-NQO and arecoline treatment for 8 weeks. The arrows indicate an enlargement of esophageal tumor. The H&E sections showed esophageal papilloma and invasive squamous cell carcinoma.

Arecoline is the most abundant of the four main alkaloids found in areca nuts and has received significant attention as a potential carcinogen.[16] Our clinical findings showed that patients with a history of areca nut chewing have a younger age of onset than those without a history of areca nut chewing. To further explore whether esophageal tumorigenesis is reinforced by areca nut use, animal models simulating the human ESCC condition are of key importance. The 4-NQO is one of the carcinogens which can induce esophageal carcinogenesis and may serve as a surrogate for tobacco exposure.[14] To validate our clinical finding, we used a 4-NQO murine ESCC model to test whether arecoline, a main constituent of areca nut, can accelerate esophageal tumorigenesis. Grossly, the esophageal lesions were usually multifocal. There were several lesions at different sites on one esophagus. The gross lesion of papilloma usually had exophytic growth appearance and papillomatous or rounded surface. The gross lesions of invasive squamous cell carcinomas usually had ulcerated and uneven surface with elevated and indurated border. Among the 16 mice exposed to 4-NQO only for 16 weeks, 14 survived at the end of 28 weeks, and 11 (79%) and 7 (50%) of the 14 mice had the esophageal papilloma and invasive squamous cell carcinoma, respectively. Among the 16 mice exposed to concomitant 4-NQO and arecoline treatment for 16 weeks, 13 survived at the end of 28 weeks, and 12 (92%) and 9 (69%) of the 13 mice had esophageal papilloma and invasive squamous cell carcinoma, respectively. We did not observe significant difference in the incidence of esophageal papilloma or invasive squamous cell carcinoma between mice exposed to concomitant 4-NQO and arecoline treatment for 16 weeks and mice exposed to 4-NQO only for 16 weeks (Table 4). However, the incidence of esophageal papilloma in mice exposed to concomitant 4-NQO and arecoline treatment for 8 weeks was significantly higher than that in mice exposed to 4-NQO only for 8 weeks (67% versus 31%; P = 0.049; Table 4 and Fig 2). The incidence of esophageal invasive squamous cell carcinoma in mice exposed to concomitant 4-NQO and arecoline treatment for 8 weeks was also significantly higher than that in mice exposed to 4-NQO only for 8 weeks (40% versus 6%; P = 0.037; Table 4 and Fig 2). Among the 16 mice exposed to 4-NQO only for 8 weeks, 5 (31%) and 1 (6%) had the esophageal papilloma and invasive squamous cell carcinoma, respectively. Among the 16 mice exposed to concomitant 4-NQO and arecoline treatment for 8 weeks, 15 survived at the end of 28 weeks, and 10 (67%) and 6 (40%) of the 15 mice had esophageal papilloma and invasive squamous cell carcinoma, respectively.

**Table 4:** Comparison of the incidence of esophageal papilloma and invasive SCC in mouse model.

Group	4-NQO	Arecoline	Weeks of 4-NQO/Arecoline treatment	No. of mice at week 0	No. of mice surviving at 28 weeks	Incidence of esophageal papilloma	Incidence of sophageal Invasive SCC
1	100μg /mL	0	16	16	14	79% (11/14)	50% (7/14)
2	100μg/mL	500μg/mL	16	16	13	92% (12/13)	69% (9/13)
3	100μg /mL	0	8	16	16	31% (5/16)^a^	6% (1/16)^b^
4	100μg/mL	500μg/mL	8	16	15	67% (10/15)^a^	40% (6/15)^b^
5	0	500μg/mL	16	8	8	0% (0/8)	0% (0/8)
6	0	0	0	8	8	0% (0/8)	0% (0/8)

4-NQO, 4-nitroquinoline 1-oxide; SCC, squamous cell carcinoma

^*a*^ Significant difference (P = 0.049) in the incidence of esophageal papilloma between group 3 and group 4.

^b^ Significant difference (P = 0.037) in the incidence of esophageal invasive SCC between group 3 and group 4.

Chi-square or Fisher's exact test was used for statistically analysis.

### The correlation between p53 immunohistochemical staining and areca nut chewing

**Fig 3:**
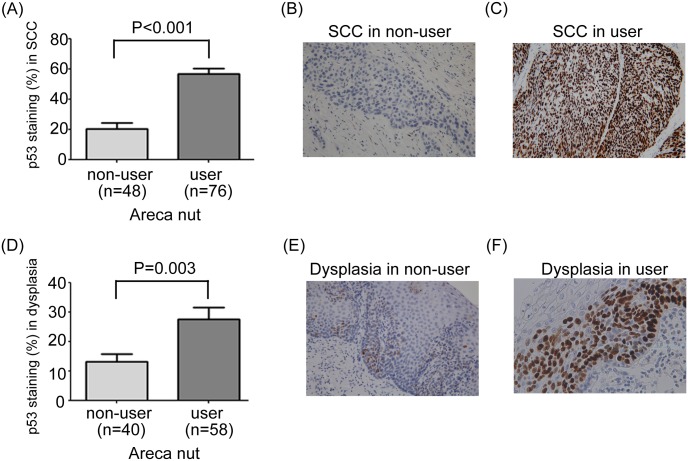
(A) The percentage of p53 immunohistochemical staining in esophageal squamous cell carcinoma in patients with areca nut chewing history was significantly higher than that in patients without areca nut chewing history. (B) Representative example of low p53 immunohistochemical staining in squamous cell carcinoma in a patient without areca nut chewing history. Original magnification X 200. (C) Representative example of high p53 immunohistochemical staining in squamous cell carcinoma in a patient with areca nut chewing history. Original magnification X 200. (D) The percentage of p53 immunohistochemical staining in esophageal dysplasia in patients with areca nut chewing history was significantly higher than that in patients without areca nut chewing history. (E) Representative example of low p53 immunohistochemical staining in dysplasia in a patient without areca nut chewing history. Original magnification X 200. (F) Representative example of high p53 immunohistochemical staining in dysplasia in a patient with areca nut chewing history. Original magnification X 400. SCC, squamous cell carcinoma.

Previous studies[17, 18] revealed that there was a correlation between TP53 gene status and the response to chemoradiotherapy in ESCC patients. Immunohistochemical staining is an accessible method and a good surrogate of TP53 gene status. To compare the p53 immunohistochemical staining in the tumor and dysplasia between patients with and without a history of areca nut chewing, we performed p53 immunohistochemistry in esophagectomy specimens in patients receiving surgery. Among 129 patients receiving surgery, paraffin blocks were available in 124 patients, and 98 of the 124 patients had dysplasia simutaneously. For p53 immunohistochemical staining in squamous cell carcinoma, patients with areca nut chewing history had significantly more nuclear staining than those without areca nut chewing history (57% versus 20%; P<0.001; Fig 3A–3C). For p53 immunohistochemical staining in dysplasia, patients with areca nut chewing history also had significantly more nuclear staining than those without areca nut chewing history (28% versus 13%; P = 0.003; Fig 3D–3F).

## Discussion

In the present study, we observed significantly inferior OS rates in ESCC patients with a history of areca nut chewing. To the best of our knowledge, only one previous study has investigated areca nut chewing as a prognostic factor for ESCC.[13] In that report, Wu *et al*. also found that areca nut chewing is associated with worse survival in patients with ESCC with the mean survival in patients with and without areca nut chewing being 17.8 and 24 months, respectively. However, the distribution of treatment modalities and the interactions between areca nut chewing and treatment modalities were not described in their study. In our study, we collected detailed information regarding treatment modalities and performed a subgroup analysis of each treatment group. Among the patients receiving surgery, there was no significant association between areca nut chewing and survival. Interestingly, however, we found a significant impact of areca nut chewing among the patients who underwent preoperative chemoradiotherapy. The 3-year OS rate was 24% in patients with areca nut chewing history, compared with 50% in patients without areca nut chewing history. To further explore why the patients with areca nut chewing history who underwent preoperative chemoradiotherapy had worse treatment outcomes, the response to preoperative chemoradiotherapy among the patients with and without areca nut chewing history was analyzed and compared. We found that the pCR rate after preoperative chemoradiotherapy was significantly lower in patients with areca nut chewing history than in patients without such history (21% versus 44%; P = 0.002). These results suggest that areca nut chewing might affect sensitivity to chemoradiotherapy.

The explanation why a history of areca nut chewing is associated with poor response to preoperative chemoradiotherapy remains unclear. Mutations of the TP53 gene resulting from areca nut chewing might be one possibility. Makino *et al*.[17] found that the presence of TP53 gene mutations was associated with non-pCR after chemoradiotherapy in ESCC patients. A recent meta-analysis performed by Zhang *et al*.[18] also revealed that ESCC patients with wild-type TP53 gene status had a higher pCR rate to neoadjuvant chemoradiotherapy. In our study, we found that patients with areca nut chewing history had significantly more p53 immunohistochemical staining than those without areca nut chewing history, suggesting the association between areca nut chewing and the presence of TP53 gene mutation.. Goan *et al*.[19] also reported that ESCC in areca nut chewers exhibited a significantly higher incidence of TP53 gene mutations than in non-chewers (67.6% versus 32.4%, P = 0.007). These reports may explain the poor response to chemoradiotherapy among patients with a history of areca nut chewing in the present study. Further studies are necessary, however, to investigate the associations between areca nut chewing, TP53 gene status, and the response to chemoradiotherapy in patients with ESCC.

In our study, the patients with areca nut chewing history had a significantly younger age of onset than those without areca nut chewing history (52.68-year-old versus 56.75-year-old; P<0.001). A previous study conducted by Lin *et al*.[12] also found an earlier age of onset (6.34 years younger) for ESCC among areca nut chewers than non-chewers. These results suggest that areca nut usage plays an important role in accelerating the carcinogenesis of the esophagus. Furthermore, the results of our animal experiments also supported the clinical observation. We found that the incidence of esophageal invasive squamous cell carcinoma in mice administered 100μg /mL of 4-NQO for 8 weeks was 6% at the end of 28 weeks. However, when 100μg /mL 4-NQO was combined with 500μg /mL arecoline, the incidence of esophageal invasive squamous cell carcinoma increased to 40% by 28 weeks. Nontheless, the mechanism of tumorigenesis promotion by arecoline in 4-NQO-induced murine ESCC remains unclear. Previous studies[20, 21] have reported that arecoline could repress DNA repair ability and inhibit p53, and these effects may contribute to the arecoline's promotion of tumor development.

Our study has several limitations. First, the present study was a retrospective analysis. Histories of smoking, alcohol use, and areca nut chewing were obtained from medical charts. We did not have questionnaires for these patients to collect further data. Furthermore, our observations were based on a relatively small number of patients.

## Conclusions

In conclusion, our results indicate that areca nut chewing history is significantly associated with a younger age of onset, poor response to chemoradiotherapy, and shorter overall survival in patients with ESCC. Arecoline, a main constituent of areca nut, accelerates esophageal tumorigenesis in 4-NQO-induced murine ESCC model. Further studies are necessary to validate our results and evaluate the underlying mechanism of different sensitivities to chemoradiotherapy between patients with and without a history of areca nut chewing.

## Abbreviations

4-NQO: 4-nitroquinoline 1-oxide
AJCC: American Joint Committee on Cancer\
ESCC: esophageal squamous cell carcinoma
H&E: Hematoxylin and eosin stained
OS: Overall survival
pCR: pathologic complete response
